# Chaos-Enhanced, Optimization-Based Interpretable Classification Model and Performance Evaluation in Food Drying

**DOI:** 10.3390/biomimetics11010078

**Published:** 2026-01-18

**Authors:** Cagri Kaymak, Bilal Alatas, Suna Yildirim, Ebru Akpinar, Gizem Gul Katircioglu, Murat Catalkaya, Orhan E. Akay, Mehmet Das

**Affiliations:** 1 Department of Mechatronics Engineering, Faculty of Engineering, Firat University, Elazig 23119, Turkey; 2 Department of Software Engineering, Faculty of Engineering, Firat University, Elazig 23119, Turkey; 3 Department of Software Engineering, Faculty of Engineering and Natural Sciences, Malatya Turgut Ozal University, Malatya 44920, Turkey; 4 Department of Mechanical Engineering, Faculty of Engineering, Firat University, Elazig 23119, Turkey; 5 Gas and Installation Technology Department, Technical Sciences Vocational School, Kahramanmaras Sutcu Imam University, Kahramanmaras 46050, Turkey; 6 Department of Mechanical Engineering, Faculty of Engineering and Architecture, Kahramanmaras Sutcu Imam University, Kahramanmaras 46000, Turkey

**Keywords:** smart food drying, explainable artificial intelligence, rule mining, energy efficiency, sunflower optimization algorithm

## Abstract

Food drying is a widely used preservation technique; however, achieving high energy efficiency while maintaining product quality remains a significant challenge. This study aims to analyze comprehensive experimental data obtained during the hot-air drying process of the Paşa pear (regional pear) and the system’s autonomous control structure using an explainable artificial intelligence (XAI)-based method. The intelligent drying system, operating for approximately 17.5 h under two temperatures (50 °C and 65 °C) and two air speeds (0.63 m/s and 1.03 m/s), continuously adjusted the temperature and air speed using a PLC-based control mechanism; it ensured stable control throughout the process by monitoring parameters such as product weight, moisture, inlet–outlet temperatures, and air speed in real time. Experimental results showed that drying performance varied significantly with operating conditions, with product mass decreasing from 450 g to 103 g. The innovative aspect of the study is that it obtained quantitative, interpretable rules without discretization by applying the oscillatory chaotic sunflower optimization algorithm (OCSFO) to multidimensional control and process data for the first time. Thanks to its chaotic search mechanism, OCSFO accurately analyzed complex drying dynamics and created rules that achieved over 90% success for high, medium, and low performance classes. The obtained explainable rules clearly demonstrate that drying temperature and air velocity are the dominant determining parameters for drying efficiency, while energy consumption and cabin temperature distribution play a supporting role in distinguishing between efficiency classes. These rules clearly demonstrate how changes in controlled temperature and air velocity, combined with product weight and heat transfer, affect drying performance. Thus, the study offers a robust framework that identifies critical factors affecting drying performance through a transparent artificial intelligence approach that leverages both the autonomous control system and XAI-based rule mining.

## 1. Introduction

It is well known that the development of societies is directly related to people’s balanced and healthy nutrition with quality foods. For society to meet its needs and maintain a healthy lifestyle, it must have easy access to healthy foods [1].

In recent years, food technology producers, motivated by a desire to access healthier food, have encouraged the cultivation of higher-quality products. The most common method used to meet the need for seasonal food consumption is food drying. The drying process involves heat and mass transfer, and drying agricultural products with solar energy is recognized as one of the oldest food preservation methods. Sunlight food drying causes a serious decline in food quality due to environmental factors. For this reason, performing the drying process with specially designed artificial dryers both shortens the drying time and enables the production of higher-quality, more hygienic products with a longer shelf life. Thanks to food-drying systems developed with technological advances, access to food available in almost every season has been made possible. In recent years, the food drying sector has made significant progress with artificial intelligence and control techniques.

Hosseinpour et al. [2] developed a fixed, unscaled image texture processing method to eliminate the effects of sample shrinkage on texture properties during the drying of shrimp. The images of texture properties were processed using an artificial neural network (ANN) to estimate the moisture content of shrimp. Janjai et al. [3] developed an innovative approach to evaluate the effectiveness of solar drying for Chinese cherries (lychees) in a parabolic greenhouse dryer using an ANN. They conducted ten trials with 100 kg of lychees to achieve higher performance. They used an ANN model to dry lychees and model their performance. To test the success of the ANN model, they evaluated seven standard datasets to train an ANN model with four inputs, one output, and two hidden layers. As a result, they found that their method had the capacity to predict the dryer’s operations after sufficient training. Guiné et al. [4] introduced an ANN modeling technique for the antioxidant activities and phenolic components of bananas for different drying methods. The bananas were air-dried at 50–70 °C and then cleaned and lyophilized, during which they were examined for antioxidant and phenolic content. The ANN modeling they applied in their study revealed that the number of phenolic compounds and antioxidant activity could be predicted with high accuracy using simple ANNs based on drying conditions, banana varieties, and specific types. Taheri-Garavand et al. [5] found that ANN successfully predicted drying parameters (moisture ratio, dry material, energy efficiency, and exergy efficiency) during the hot-air drying process of banana slices. Liu et al. [6] preferred the Bayesian extreme learning machine (BELM) method to analyze the color changes of mushroom slices throughout the drying process. In their research, they found that BELM predicted color changes in mushroom slices during drying more effectively than classical backpropagation neural networks. Nadian et al. [7] developed an intelligent fuzzy artificial vision control system that combines computer vision with fuzzy logic to manage process parameters in complex hot air and infrared drying processes, monitor color and volume changes during kiwi drying, and improve the quality of products obtained using a genetic algorithm (GA). This innovation reduced the time from 40 min to 24 min (40%) compared with the hot-air drying method alone. Chakravartula et al. [8] used a convective dryer equipped with a computer vision system and a load cell to continuously monitor unbleached or bleached carrot slices during the drying process. The setup of these systems enabled them to use the linear regression method to track product characteristics (weight, color, and size) and develop moisture prediction models related to shrinkage. The linear models based on the shrinkage examined yielded better results compared to more complex, specific thin-layer models.

All studies in the literature show that model results are mostly dependent on systems that operate as black boxes. In recent years, particularly in the fields of engineering and energy systems, the limitations of such black-box approaches have been highlighted, and it has been reported that interest in explainable artificial intelligence (XAI) methods, which aim to increase the understandability of decision mechanisms, has rapidly increased [9,10].

In recent years, optimization algorithms inspired by biomimetics have garnered significant attention in energy systems, process optimization, and intelligent control applications [11,12]. These methodologies, which replicate natural phenomena like swarm behavior, evolutionary adaptation, and plant intelligence, provide effective search capabilities for addressing difficult and nonlinear optimization challenges. Bio- and nature-inspired optimization algorithms have been effectively utilized in energy-efficient system design, renewable energy management, thermal system design, and control parameter tuning, where traditional deterministic methods often underperform [13,14,15].

Additionally, explainable artificial intelligence (XAI) has arisen as a pivotal research focus in engineering applications, seeking to address the shortcomings of opaque learning models by offering transparent and interpretable decision-making processes [16,17,18,19]. Recent studies have shown that combining bio-inspired optimization algorithms with interpretable models can markedly improve decision transparency, model dependability, and physical interpretability [11,12,13,14,20,21,22]. Notwithstanding these advancements, the amalgamation of plant intelligence-based optimization algorithms with XAI-driven quantitative rule mining for food drying systems remains predominantly unexamined. However, the model obtained in this study consists of explainable, understandable, transparent, and interpretable rules, and the effects of the relevant input parameters and their appropriate ranges on the output parameter are clearly visible. The sunflower optimization (SFO) algorithm is a nature-inspired optimization algorithm developed based on the sun-seeking behavior of sunflowers. The fundamental principle of the algorithm is based on iteratively moving towards the best solution, referred to as the “sun”. Although it is a relatively new method in the literature, it has produced successful results in various engineering problems. Qais et al. [23] used SFO for modeling and simulating photovoltaic modules and demonstrated that high accuracy could be achieved even with standard algorithm parameters. Gomes et al. [24] compared SFO with genetic algorithms and improved SFO variants in the damage detection problem of plate-like structures and reported that SFO-based approaches exhibited superior performance. Yuan et al. [25] proposed an improved SFO with a self-adaptive weighting mechanism for the parameter optimization of proton exchange membrane fuel cells (PEMFC). Hussein et al. [26] used SFO for optimizing PI controller parameters and compared the results with particle swarm optimization. Shaheen et al. [27] and Alshammari et al. [28] demonstrated that chaotic map-based SFO variants are more successful than classical optimization algorithms in the field of power systems. However, the vast majority of existing SFO-based studies focus on continuous optimization and black-box performance improvement problems; the potential of SFO in interpretable decision support systems, such as XAI and rule mining, has not yet been sufficiently addressed in the literature.

An artificial intelligence model that controls both the drying process and system energy and has explainable, interpretable features has not been used in food drying systems. In this study, an intelligent drying system capable of autonomously adjusting temperature and air speed for drying the “Paşa” pear, a variety specific to Elazig, was developed, and various drying scenarios were created. A total of four experiments, involving two different temperatures (50 °C and 65 °C) and two different air speeds (0.63 m/s and 1.03 m/s), lasted approximately 17.5 h in the designed system, and data were collected from the system at one-minute intervals. In the experiments, the product’s weight, surface temperature, inlet and outlet air temperature and humidity values, cabin internal temperature, and air speed were continuously measured, and energy consumption was analyzed. The experimental data were analyzed using explainable artificial intelligence (a hybrid method based on the sunflower optimization (SFO) algorithm), and the effects of drying parameters on energy consumption and drying efficiency were determined using transparent and interpretable rules. Thanks to these rules, optimal drying conditions were identified, and high-quality products were obtained with minimal energy consumption.

## 2. Materials and Methods

### 2.1. Experimental Drying System and Hardware Configuration

**Figure 1:**
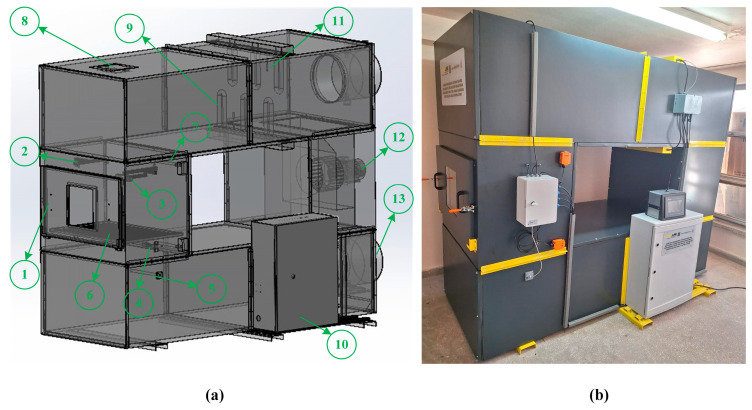
Smart drying system: (**a**) three-dimensional design; (**b**) final state of the manufactured system.

Figure 1a shows the three-dimensional design of the system designed for the experiments conducted and the numbering of its components, while Figure 1b shows the final state of the manufactured system.

The list of hardware components numbered in the three-dimensional design in Figure 1a is provided in Table 1.

**Figure 2:**
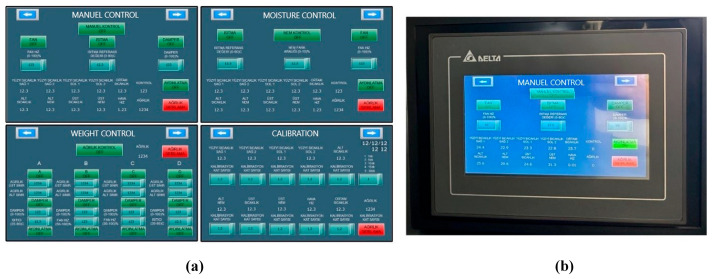
HMI panel interface screens: (**a**) interface designs; (**b**) interface example on the system.

When food samples are tested by drying, interface pages are created on the PLC’s HMI panel to enable the necessary calibrations and controls to be performed so that parameters such as temperature, humidity, weight, and air velocity can be successfully measured. This was implemented on the system as shown in Figure 2.

### 2.2. Experimental Procedure and Drying Scenarios

**Figure 3:**
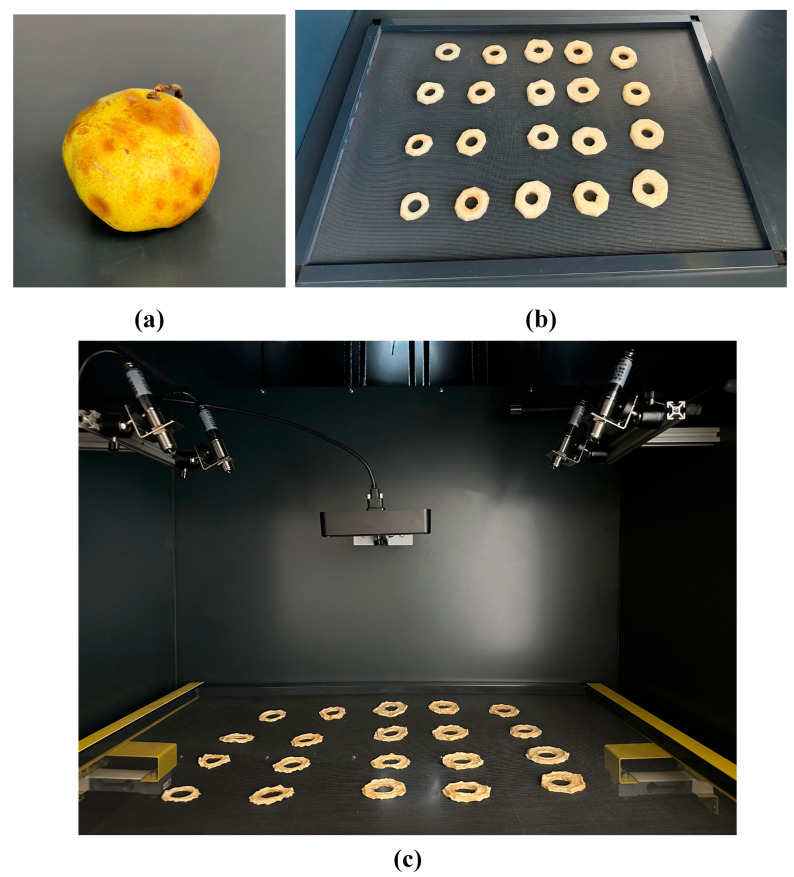
Cabin interior view and food sample for drying: (**a**) food sample (Paşa pear); (**b**) drying tray; (**c**) inside the drying cabinet.

In the drying experiments conducted, the pear product was cut into oval pieces that were 10 mm thick. The fruit seeds were removed and placed on the drying tray. The tray had 0.5 mm holes, and its instantaneous weight was measured using load cells with a capacity of 10 kg on both sides. In the drying experiments, fresh air was supplied in a closed cycle, and when the humidity of the inlet and outlet air was equalized, fresh air was supplied again with the help of automatic open–close flaps. During the food-drying process, the system included 4 IR temperature sensors measuring the product surface temperature, 2 load cells measuring the product weight, air temperature, and humidity sensors at the drying compartment inlet and outlet, a cabin internal temperature sensor, and transmitter sensors measuring the drying air velocity. In the experiments conducted, air speeds of 0.63 m/s and 1.03 m/s were initially selected. Experiments were conducted at drying temperatures of 50 °C and 65 °C for each speed value. In line with the drying scenarios and quality constraints, a drying process with optimal energy use and high product quality was established. The initial and final states of the dried products are shown in Figure 3.

All sensor data were continuously recorded at 1-min intervals throughout the drying process. The raw data obtained were resampled at 10-min intervals to both represent the drying kinetics more consistently and to create a data structure suitable for explainable artificial intelligence (XAI)-based rule mining analyses. The resulting dataset preserved the temporal dynamics of the drying process while reducing measurement noise and enabling interpretable rule extraction.

### 2.3. Drying Kinetics and Energy Calculations

Mass transfer calculations for the product were performed in drying experiments. In the experiments, the product’s wet- and dry-basis moisture content, moisture ratio values, and drying efficiency values are calculated. The moisture content of food products is evaluated as the amount of water they contain. Percentage parameters are used to express the amount of water in food. Wet- and dry-basis definitions are used to determine moisture content. Equations (1) and (2) are used to calculate the moisture content (MC) of the food product on a wet basis (wb) and dry basis (db).

(1) $${MC}_{wb}=\frac{W_{s}}{W_{s}+W_{k}}\times 100,$$

(2) $${MC}_{db}=\frac{W_{s}}{W_{k}}.$$

In the equations, $W_{s}$ is the wet weight and $W_{k}$ is the dry weight. Dimensionless moisture ratio (MR) values were calculated using Equation (3).

(3) $$MR=\frac{M-M_{e}}{M_{o}-M_{e}}.$$

Equation (3) presents the equilibrium relative humidity value of the dried product $M_{e}$. The moisture content specified here represents the solid-matter-based reference moisture content determined by the Shimadzu MOC63u device (72.9 g water/g solid), not the environmental equilibrium moisture content. In Equation (4), $\eta_{d}\;$ is the dryer efficiency, $m_{i}\;$ and $m_{f}$ are the initial and final masses of the pear product (kg), $L_{w}$ is the latent heat of vaporization (kJ/kg), $C_{pw}$ is the specific heat of water (kJ/kg. K), $T_{o}$ and $T_{a}$ are the dryer outlet temperature and ambient temperature (K), respectively, and $E_{T}$ is the total energy consumed by the heater and fan in the system.

(4) $$\eta_{d}=\frac{{(m}_{i}-m_{f})\times (L_{w}+C_{pw}(T_{o}-T_{a}))\;\;}{M_{o}-M_{e}}\times 100\%.$$

### 2.4. Measurement Uncertainty Analysis

In the measurements performed, the equation provided by Holman et al. [29], expressed in Equation (5), was used for uncertainty analysis. In uncertainty analysis, also known as the partial differential method, it is expressed as the total error ratio (WR) in a measurement with n independent variables.

(5) $$WR={\left[{\left(\frac{\partial R}{{\partial x}_{1}}W_{1}\right)}^{2}+{\left(\frac{\partial R}{{\partial x}_{2}}W_{2}\right)}^{2}+{\left(\frac{\partial R}{{\partial x}_{3}}W_{3}\right)}^{2}+\cdots +{\left(\frac{\partial R}{{\partial x}_{n}}W_{n}\right)}^{2}\right]}^{1/2}.$$

In Equation (5), R represents the dimension to be measured, $x_{1},x_{2},x_{3},\ldots ,x_{n}$ represent the parameters affecting the measurement, and $W_{1}$, $W_{2}$, $W_{3}$,…$W_{3}$ represent the error rate related to the independent variable [30]. The uncertainty values calculated for the measurement parameters are presented in Table 2.

### 2.5. Input and Output Variables for Explainable AI (XAI) Modeling

In this study, the dataset used for the XAI-based rule mining model is structured with seven input variables and one output variable obtained from experimental measurements. The input variables represent the thermal, fluid, and energy-based dynamics of the drying process, while the output variable indicates the classified state of drying efficiency. The model’s output variable consists of high (H), medium (M), and low (L) classes defined according to drying efficiency values. The parameters used in the rules generated by explainable artificial intelligence methods for the pear product are given in Table 3.

***Meaningfulness of Parameters and Literature Support***

***Output and input temperatures*** ($T_{O}$, $T_{I}$): Directly affect moisture removal rate. Temperatures outside the optimal range may cause crusting on the product surface or excessive energy consumption [2].

***Air velocity (***V***)***: High air velocities accelerate drying by increasing the moisture transfer coefficient. However, very high velocities can increase energy consumption [3].

***Product weight (***G***)***: Weight change is directly related to the decrease in moisture content. Mass loss reflects the degree of progress in the drying process [7].

***Heat energy produced (***$Q_{U}$***)***: This is directly related to the energy expended in the drying cabinet for the product’s moisture loss. Energy efficiency increases within the optimal range [1].

***Total energy consumption (***$E_{T}$***)***: Efficiency is a directly effective parameter, as it is the ratio between the energy supplied to the system and the moisture removed [5].

### 2.6. Class Definition and Labeling for Drying Efficiency

This study regarded drying efficiency as a categorical output variable to facilitate explainable rule-based modeling. According to the computed drying efficiency values and quality–energy trade-off assessments, the drying process was categorized into three distinct performance levels: high (H), medium (M), and low (L) efficiency. This class-based format facilitates the derivation of clear and interpretable decision rules, explicitly demonstrating how varying operational parameter ranges affect drying performance. The implemented labeling technique guarantees an equitable depiction of efficiency levels while maintaining the physical significance of the drying process, thus enabling dependable explainable artificial intelligence (XAI) analysis.

In this study, drying efficiency was treated as a categorical output variable for explainable rule-based modelling. The drying efficiency values calculated from the experiments have been divided into three separate performance levels, taking into account both the numerical distribution and the energy–product quality balance: high (H), medium (M), and low (L). The high-efficiency class represents operating conditions in which maximum moisture removal per unit of energy is achieved while maintaining product quality. The medium-efficiency class denotes situations in which the system exhibits acceptable performance but is only partially deviating from optimal conditions. The low efficiency class covers operating conditions where moisture removal remains limited despite increased energy consumption, particularly in the final stages of drying. The threshold values for these classes have been determined based on drying efficiency ranges obtained from experimental data and are summarized in Table 4.

In this study, average drying efficiency was preferred over instantaneous drying efficiency as the classification target. Average drying efficiency is a stable performance indicator at the system level. It shows how much of the total energy input during the drying process is effectively used for moisture removal. Instantaneous drying efficiency values are highly sensitive to transient heating effects, sensor sensitivities, and short-term fluctuations, especially in the initial stages of drying. This sensitivity often makes it hard to obtain physically meaningful and generalizable rules from explainable artificial intelligence-based rule-extraction processes. In contrast, average drying efficiency is a more reliable and interpretable target for defining high (H), medium (M), and low (L) efficiency classes. It reflects the initial and final moisture conditions of the product, total energy consumption, and the energy–mass transfer balance of the entire drying process.

### 2.7. Sunflower Optimization (SFO) Algorithm

The study utilized the sunflower optimization (SFO) algorithm, a plant intelligence algorithm. There are two key reasons for choosing the SFO algorithm. First, plant intelligence-based approaches have not been previously studied for rule inference problems, to the best of our knowledge. Therefore, there is a need for a study in this field that can measure the performance of such algorithms. Second, the high performance of the SFO algorithm, which uses the internal loop principle, provides an advantage over other plant-based approaches. For these reasons, the SFO algorithm was selected to solve this problem.

At the core of the SFO algorithm lies the movement of sunflowers following sunlight [31]. In the SFO algorithm mechanism, individuals want to maintain their position if they are close to the sun and show greater orientation to approach the sun if they are far away. One of the important principles in the algorithm is the representation of sunflowers’ orientation towards the sun. The sun ($X^{*}$), which is the current best solution in the population, serves as a reference for other sunflower individuals ($X_{i}$). The orientation of the population containing $n_{p}$ individuals towards the sun is denoted by $s_{i}$ and is generally expressed as in Equation (6).

(6) $$s_{i}=\frac{X^{*}-X_{i}}{\left|\left|X^{*}-X_{i}\right|\right|}\;,\;\;\;\;\;i=1,2,\ldots ,n_{p}.\;$$

The algorithm’s progression toward the optimum is called the direction and is expressed by Equation (7). The convergence is based on two fundamental parameters. These are λ, the inertia coefficient, and $P_{i}$, which represents the probability of convergence between the i-th individual and the $\left(i-1\right)$-th individual. As can be seen from the general expression, individuals closer to the sun will converge toward it in small steps, while those farther away will attempt to converge in larger steps.

(7) $$d_{i}=\lambda P_{i}\left(\left\|X_{i}+X_{i-1}\right\|\right)\left\|X_{i}+X_{i-1}\right\|.$$

In the classical SFO algorithm, in each iteration, some individuals far from the sun are removed from the search process according to the population removal rate (O), and new individuals are generated to replace them. These new individuals join the population in each iteration, helping to find different exploration points. In addition, when determining the new positions of other individuals, it is necessary to pay attention to the maximum convergence value so as not to miss the global minimum individuals. This basic principle is provided by Equation (8). In this equation, $X_{max}$ and $X_{min}$ indicate the upper and lower limit values, while $N_{pop}$ indicates the total number of individuals in the population. Equation (9) is used to calculate the value of the new individual. These basic steps and logical functions of the SFO algorithm can be seen in the pseudocode in Algorithm 1.

(8) $$d_{max}=\frac{\left|\left|X_{max}-X_{min}\right|\right|}{2N_{pop}},$$

(9) $${\vec{X}}_{i+1}={\vec{X}}_{i}+d_{i}{\vec{s}}_{i}.$$

**Algorithm 1.** Sunflower Optimization (SFO) Algorithm pseudocode1. $X_{max},X_{min}$ ← problem-defined upper and lower bounds 2. ${Itr}_{max}$ ← maximum number of iterations 3. $N_{pop}$ ← population size 4. O ← population removal rate (%) 5. for *i* = 1 to $N_{pop}$ 6. $\;\;\;\;\;\;\;\;X_{i}=$ IndividualGeneratorFunction($X_{max},X_{min}$) 7.         AddToPopulation($X_{i}$) 8. end for 9. CalculateObjectiveValue() 10. $X^{*}$ ← FindSun() 11. OrientTowardsSun() 12. while(*i* < ${\mathrm{Itr}}_{\max}$) 13.      for *j* = 1 to $N_{pop}$ 14.              CalculateIndividualVector() 15.              RemoveDistantIndividuals(%O, *i*) 16.              EvaluateNewIndividuals($X^{*}$) 17.      end for 18.      CalculateObjectiveValue() 19.   $X^{*}$ ← FindSun() 20. End while 21. DisplayBest()


In the SFO algorithm, the inverse square law of radiation applies, and one factor is the distance from the sun. In this law, there is an exponential (usually quadratic) inverse relationship between the radiation produced and the distance to the target. In other words, the distance of a sunflower (individual solution) from the best solution (the sun) will determine its presence within the population. This effect is denoted by $R_{i}$ and is generally expressed as in Equation (10). In this equation, G is the power of the source and $r_{i}$ is the distance between the i-th individual and the current best solution.

(10) $$R_{i=}\frac{G}{{4\pi r}_{i}^{2}}.$$

While the SFO algorithm has exhibited competitive efficacy in numerous optimization challenges, its direct utilization in explainable rule mining tasks may be hindered by restricted exploration capacity and premature convergence when addressing intricate and nonlinear parameter interactions. This paper introduces an improved variation, called the oscillating chaotic sunflower optimization (OCSFO) algorithm, to address these constraints and improve global search diversity and solution stability. OCSFO enhances the original SFO framework by incorporating oscillatory search dynamics and chaotic mapping processes to improve rule diversity, convergence robustness, and interpretability in XAI rule mining applications.

### 2.8. Oscillating Chaotic Sunflower Optimization (OCSFO) Algorithm

This section provides a detailed description of the oscillating chaotic sunflower optimization (OCSFO) [32] algorithm, emphasizing the improvements made to improve exploration diversity and convergence stability. In the classical SFO algorithm, rapid and excessive population growth is undesirable. For this reason, the algorithm executes an individual elimination mechanism within the population, which depends on an elimination coefficient (mortality rate). Excessive individual elimination is prevented by adding a certain proportion of new individuals to the population. A point that is often overlooked here is the possibility that the newly generated individuals may be similar to other individuals in the population. The OCSFO algorithm, which is a hybrid of chaotic search and sunflower optimization, uses a new oscillation-based individual generation to prevent this. Thus, the newly generated individual is positioned with limited randomness relative to the population optimum and the search space limits. A two-stage process is executed for this purpose. The first stage involves determining the position of the new individual solutions between the best solution and the search limits for a predefined ratio (Y). To do this, a Gaussian Distribution-based value is used, whose mean is the average distance between individuals. The mathematical expression for this stage is as shown in Equations (11) and (12). Here, the problem size D, (f∈D), mean value $P_{f}$, location center $L_{f}$, and variance $\sigma^{2}$ are the determining parameters.

(11) $$P_{f}=\frac{X_{f}^{*}+X_{f}^{max}}{2},$$

(12) $$L_{f}=N\left(P_{f},\sigma^{2}\right).$$

The second stage in generating the individual that will represent the new solution is to prevent the individuals produced from being similar to each other. Accordingly, the new individual position is then passed through a trigonometric auxiliary function presented in Equation (13). Accordingly, the random chaotic value ɳ and a predefined constant *β* cause the new position to oscillate relative to its previous value.

(13) $$X_{f}^{new}=L_{f}\left(1+ɳcos(\frac{2\pi i}{\beta })\right).$$

Unlike the classical SFO algorithm, the OCSFO algorithm uses chaotic map functions to add new individuals to the population. This ensures that all individuals, including the initial population, are generated chaotically. The performance of the OCSFO algorithm has been tested using different chaotic map functions known in the literature. The pseudocode of the algorithm is provided in Algorithm 2. **Algorithm 2.** Oscillating Chaotic Sunflower Optimization (OCSFO) Algorithm pseudocode1. $X_{max},X_{min}$ ← define lower and upper bounds 2. ${Itr}_{max}$ ← iteration number 3. $N_{pop}$ ← population value 4. k ← which chaotic function 5. O ← elimination rate (%) 6. $\mathrm{for}\;i\;=\;1\;\mathrm{to}\;N_{pop}$ 7. $X_{i}=$$\;\mathrm{IndividualGenerator}(k,X_{max},X_{min}$) 8. $\mathrm{AddIndividual}(X_{i}$) 9. end for 10. FindObjectiveValues() 11. $X^{*}$ ← FindBest() 12. OptimizeSolutionsTowardsBest() 13. $\mathrm{While}\;(\mathrm{iteration}\;\text{<}\;{Itr}_{max}$) 14. for j = 1 to … 15. FindVectorForIndividual() 16. PerformElimination(%O, %Y, i,σ
β) 17. $\mathrm{EvaluateNewlyAddedIndividuals}(X^{*}$) 18. end for 19. FindObjectiveValues() 20. $X^{*}$ ← FindBest() 21. End while 22. ReturnBestValue()where k represents the chaotic map function used. This function uses the parameters i, Y, σ, and β.

### 2.9. Hybrid Optimization-Based Quantitative Rule Mining Framework

This section presents a hybrid optimization-based quantitative rule mining framework, wherein the OCSFO algorithm is utilized to derive interpretable classification rules directly from numerical data without the need for discretization. It focuses on the applicability of the SFO algorithm, which has not been used in rule mining before, to rule mining. However, similar to other applications in the literature, the standard SFO algorithm is not directly applicable to rule inference systems, and it is thought that a version of the SFO algorithm adapted for this purpose, which is aimed at continuous optimization problems, is needed. For this adaptation, the representation forms of rule inference, individuals must be correctly determined.

There are many methods and algorithms used for classification rule mining [33,34]. Most of these classification algorithms and models are black-box-based approaches. However, understandable rules are quite important in explainable artificial intelligence data mining, which aims to arrive at correct rules as well as in datasets. Furthermore, mining interesting, understandable, and accurate classification rules in a dataset consisting of numerical features is more complex. Classification algorithms for quantitative data perform a type of discretization by performing a preprocessing step that can cause information loss. In this case, the dataset problem is changed in data mining. Therefore, the discovered classification rules belong to the model of the modified dataset. Changing the dataset is error-prone and also requires computational load. When the dataset is changed, the accuracy of the classification problem for which the solution is desired will also decrease. For this reason, it is more logical to adapt the classification method without changing the dataset [35]. Finding relevant intervals for quantitative features and combining mining for high-quality quantitative classification rules with multiple targets in a single step appear to be very meaningful in terms of speed and accuracy.

This study has adopted a different representation form for each solution in a population of n individuals in quantitative rule inference problems [35]. In this representation, a d (number of attributes) dimensional solution $X_{i}$ contains three sub-datasets: $X_{i}^{b}$, $X_{i}^{a}$, and $X_{i}^{u}(i=1,2,\ldots ,n)$. $X_{i}^{b}=(x_{1}^{b},x_{2}^{b},\ldots ,x_{j}^{b},\ldots ,x_{d}^{b})$ holds binary data for each attribute of a solution. Whether a feature $x_{j}$ is added to the solution’s rule is determined by the value $x_{j}^{b}$. In the initial population, $x_{j}^{b}$ is determined by the t(.) random generator function and a predefined threshold value “δ”. This operation is calculated using Equation (14).

(14) $$x_{j}=\left\{\begin{array}{c} 1\;\left(if\;x_{j}\;is\;used\;in\;the\;rule\right)\;\;\;\;\;\;\;if\;x_{j}^{b}>\delta ,\;\;\delta \;\epsilon \;\left[0,1\right]\;\\ 0\;\;\left(if\;x_{j}\;is\;not\;used\;in\;the\;rule\right)\;\;\;\;else \end{array}.\right.$$

$X_{i}^{a}=(x_{1}^{a},x_{2}^{a},..x_{j}^{a},..x_{d}^{a})$ represents the lower limits calculated for each property of the rule belonging to a solution, while $X_{i}^{u}=(x_{1}^{u},x_{2}^{u},..x_{j}^{u},..x_{d}^{u})$ indicates the upper limits of the attributes in the corresponding solution rule (${x_{d}^{a},x}_{d}^{u}$ ϵ R). For attribute “j” with a lower value $N^{a}$ and upper value $N^{u}$ in the search space, the condition “$N^{a}{\leq x}_{j}^{a}<x_{j}^{u}\leq N^{u}$” always holds in each iteration. During iterations, each individual recalculates its own $X_{i}^{b},X_{i}^{a}$, and $X_{i}^{u}$ values. At the end of iterations, each attribute satisfying the condition $x_{j}^{b}>\delta$ is added to the “$X_{i}$” solution rule [32]. Rule mining optimization was performed separately for each class in the dataset. This means that the main dataset (search space) is divided into subspaces for each class, and each optimization process takes place in the relevant subspace. The values of $X_{i}^{b},X_{i}^{a}$, and $X_{i}^{u}$ for a solution are checked for each data point in the relevant search space and used to calculate the fitness value of the objective function. For example, in a rule mining optimization process performed for a class “S”, assume that a solution uses the 1st, 3rd, and 5th attributes in its own rule ($x_{1}^{b},x_{3}^{b},ve\;x_{5}^{b}>\delta ,$ in iteration i-th). In this case, the individual solution will be checked for the “k” data ($X_{k_{1}},X_{k_{2}},\ldots ,X_{k_{m}}$), according to Equation (15).

(15) $$if\;x_{1}^{a}\leq \;X_{k_{1}}\leq \;x_{1}^{u}\;and\;x_{3}^{a}\leq \;X_{k_{3}}\leq \;x_{3}^{u}\;and\;x_{5}^{a}\leq \;X_{k_{5}}\leq \;x_{5}^{u}\;then\;S.$$

This calculation process is used to determine the suitability value of the objective. In the study, the “accuracy” value of the classification is taken as the objective function. To find the “accuracy” value of the $X_{i}$ solution, the condition in Equation (15) is checked individually for each data point belonging to the relevant “S” class. As a result of these operations, true positive (TP), true negative (TN), false positive (FP), and false negative (FN) values are obtained. For this purpose, as shown in Table 5, the “if” part (antecedent) and the “then” part (consequent) of the relevant rule are evaluated separately. Then, the “accuracy” value used as the objective function is calculated as shown in Equation (16) based on the TP, TN, FP, and FN values of the relevant rule.

(16) $$Accuracy=\frac{TP+TN}{TP+TN+FP+FN}.$$

TP is the number of cases where both the left and right sides of the rule are correct, TN is the number of cases where both sides of the rule are incorrect, FP is the number of cases where the left side of the rule is correct and the right side is incorrect, and FN is the number of cases where the left side of the rule is incorrect and the right side is correct. The pseudocode given in Algorithm 3 shows the operation of the processes described above in the rule-inference-based SFO algorithm [32]. The algorithm operates in two stages. The first is the training stage. In this stage, the SFO algorithm adapted according to the suitability value for the training datasets is run. The rules obtained at the end of the algorithm iterations are retested on the test data. The same evaluation processes are performed on the test data this time.
**Algorithm 3.** Adapted SFO Algorithm pseudocode1. Get the dataset 2. Initialize the encoded population showing the individual classification rule set 3. ($initialize\;X_{i}^{b},\;X_{i}^{a},\;and\;{X_{i}^{u}}^{\prime }\;for\;X_{i}$) 4. ${Itr}_{max}$ ← maximum number of iterations 5. $N_{pop}$ ← population size 6. O ← Population removal rate (%) 7. for i = 1 to $N_{pop}$ 8. $X_{i}=$ IndividualGeneratorFunction($X_{i}^{b},X_{i}^{a},X_{i}^{u}$) 9. AddToPopulation($X_{i}$) 10. end for 11. SetDefaultValues(TP, TN, FP, FN) 12. CalculateObjectiveValue() 13. $X^{*}$ ← FindSunRule() 14. while(i < ${Itr}_{max}$) 15. DirectTowardsSun() 16. for j = 1 to $N_{pop}$            a. CalculateIndividualVector()            b. RemoveDistantIndividuals(%O, i)
           c. EvaluateNewIndividuals(
$X^{*}$)
17. end for 18. SetDefaultValues(TP, TN, FP, FN) 19. CalculateObjectiveValue() 20. $X^{*}$ ← FindSunRule() 21. End while 22. TestRulesForTestData() 23. ShowBestRule()

## 3. Results

This section delineates the experimental drying outcomes, the optimization efficacy of the OCSFO algorithm, and the elucidated categorization rules derived for drying efficiency. The physical drying characteristics of “Paşa” pears under various operational settings are presented. The optimization capacity of OCSFO and the resultant interpretable rules are then shown.

### 3.1. Experimental Drying Results

**Figure 4:**
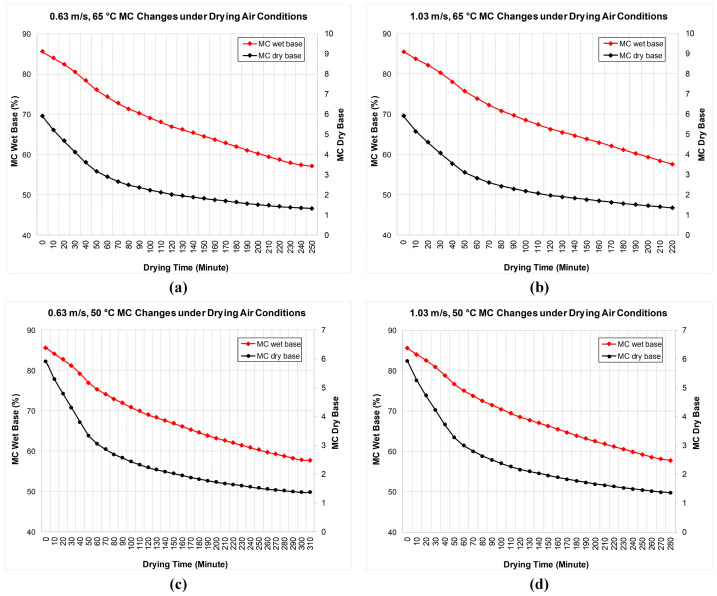
Product moisture contents (MC) under different experimental conditions: (**a**) 0.63 m/s and 65 °C; (**b**) 1.03 m/s and 65 °C; (**c**) 0.63 m/s and 50 °C; (**d**) 1.03 m/s and 50 °C.

The moisture values calculated after the experiments are shown in Figure 4. Examining Figure 4a,b, it is observed that an increase in drying air velocity shortens the drying time. At the same temperature, the liquid inside the product evaporated more quickly from the product surface. Thus, the product dried approximately 30 min earlier. Under the specified drying conditions, the wet-basis moisture content of the pear product decreased from approximately 85% to 56%. The dry-basis moisture content decreased from approximately 5.9 to 1.32. When Figure 4c,d is examined, the drying time has decreases with increasing drying air velocity. In addition, the drying time also decreased with the decrease in temperature. At the same temperature, the liquid inside the product evaporated more quickly from the product surface. Reducing the drying temperature from 65 °C to 50 °C resulted in a longer drying duration and slower moisture removal kinetics, rather than an increase in the instantaneous liquid loss rate. With the increase in air velocity, the product dried approximately 30 min earlier, while with the decrease in temperature, the drying process took an average of 75 min longer.

The MR values presented here represent a dimensionless moisture content and should not be directly correlated with the weight change criterion (Δm<1 g) used to experimentally terminate the drying process.

The time axis in each sub-graph represents the actual drying time under the relevant experimental conditions, and total drying times vary due to different air velocity and temperature values.

**Figure 5:**
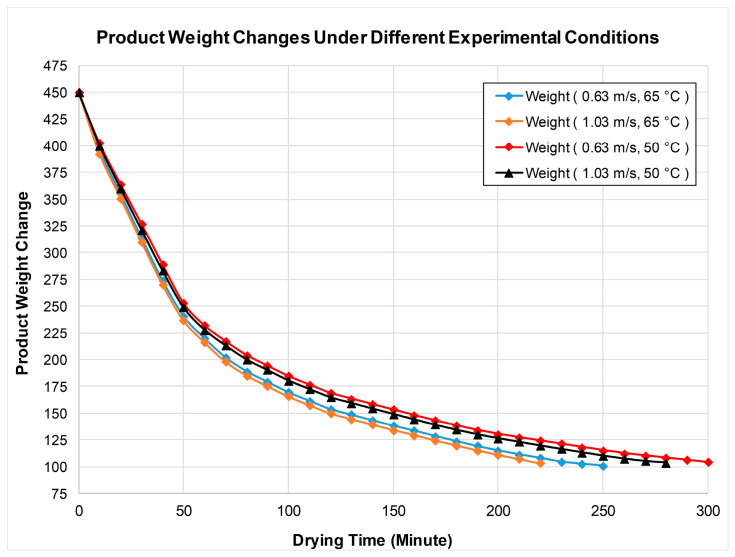
Product weight changes under different experimental conditions.

The product weight changes during the drying process under the four different experimental conditions are shown in Figure 5. The initial product weight was approximately 450 g, and the drying process continued until it reached 103 g. The experiments were terminated when the product weight change fell below 1 g. The fastest drying process occurred in the experiment conducted at an air speed of 1.03 m/s and an air temperature of 65 °C. However, the slowest drying process occurred in the drying experiment conducted at a speed of 0.63 m/s and a temperature of 50 °C.

**Figure 6:**
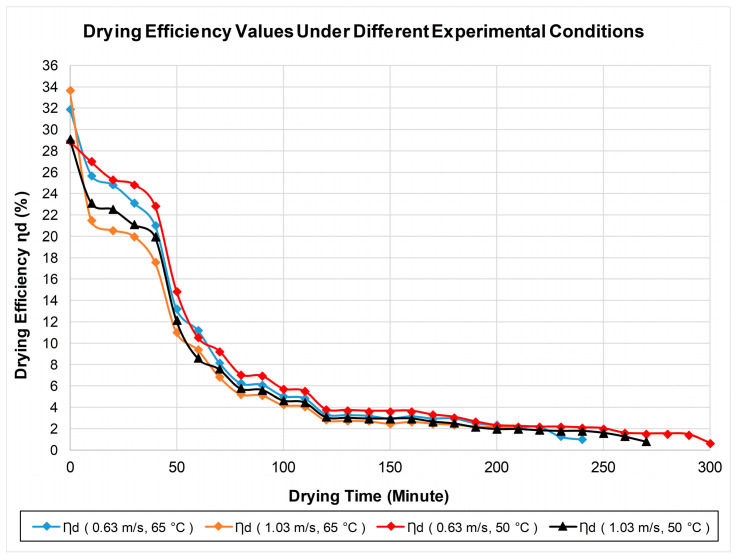
Change in drying efficiency ($\eta_{d}$) values over time.

Figure 6 shows the drying efficiency values for the pear product under four different drying conditions. Drying efficiency is calculated as the ratio of the energy required to remove liquid from the product to the total energy consumed by the system. The best average drying efficiency in this case is 8.57%, achieved at an air velocity of 0.63 m/s and an air temperature of 65 °C. Similarly, the average drying efficiency was 3.65% for the process with an air velocity of 0.63 m/s and an air temperature of 50 °C.

**Figure 7:**
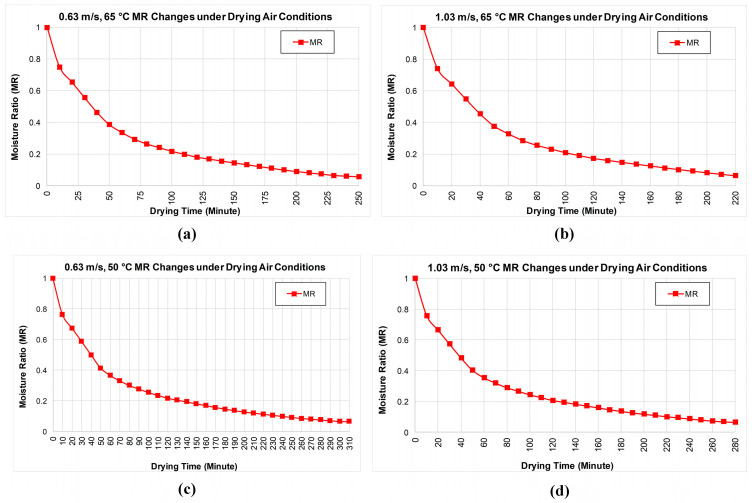
Product moisture ratio (MR) change values under different experimental conditions: (**a**) 0.63 m/s and 65 °C; (**b**) 1.03 m/s and 65 °C; (**c**) 0.63 m/s and 50 °C; (**d**) 1.03 m/s and 50 °C.

Figure 7 summarizes the moisture ratio (MR) decay for the four operating cases, showing a consistently faster drop at higher air velocity and temperature. Figure 7a (0.63 m/s, 65 °C) shows that the MR decreases from 1.00 at the start to approximately 0.39 after 50 min, 0.21 after 100 min, 0.14 after 150 min, and 0.06 after 250 min. Figure 7b (1.03 m/s, 65 °C) shows a more rapid reduction throughout the process, with MR falling to approximately 0.45 after 40 min, 0.33 after 60 min, 0.21 after 100 min, approximately 0.10 after 180 min, and approximately 0.06 after 220 min.

This indicates the shortest overall drying time among the cases. Figure 7c (0.63 m/s, 50 °C) shows a shift to longer times: MR is approximately 0.36 after 60 min, around 0.20 after 120 min, approximately 0.10 after 240 min, and reaches approximately 0.06 only after 310 min. Finally, Figure 7d (1.03 m/s at 50 °C) lies between the two 0.63 m/s cases and the 65 –1.03 m/s case. Here, MR is around 0.40 after 50 min, 0.25 after 100 min, 0.12 after 200 min, and 0.07 after 280 min. This shows that, while increasing the air velocity partly compensates for the lower temperature, it does not fully match the kinetics observed at 65 °C.

### 3.2. Performance Evaluation of the OCSFO Algorithm

In this section, the optimization performance of the OCSFO algorithm is evaluated through multifaceted analyses, including benchmark test functions and problem-specific performance metrics.

#### 3.2.1. Performance on Benchmark Test Functions

To observe the performance of the OCSFO algorithm, the test functions listed in Table 6 were chosen. Two of the test functions used are constrained (Rosenbrock Cubic/Line and Disk). The problem sizes were selected as five for Sphere, three for Rastrigin, and two for the others. The internal and solution iteration counts were 20 and 5, respectively. The dusting rate was 0.05, and the elimination rate was 0.1. In the test results for the 20 independent experiments conducted, the minimum results in the iterations were first examined. In the evaluations, both classical and hybrid algorithms were evaluated together for each test function. The quality test functions used are presented in Table 7.

Comparative statistical evaluations of independent experiments are shown in Table 8.

The results presented in the table, when evaluated according to the relevant objective functions, show that while the methods generally yield good results for Camel, which has the lowest value of −1.03, chebyshevOCSFO, logisticOCSFO, sineOCSFO, and tentOCSFO are observed to be more successful. ClassicalSFO and circleOCSFO yielded poorer results in terms of performance when considering the standard deviation and mean values. The minimum value of the nonlinear function selected as the second test function is −0.28, and all methods studied yielded extremely successful results for this function. All methods used for all tests yielded results with almost the same success rate. In the Rastrigin function, it was observed that the best result was provided by logisticOCSFO. The logisticOCSFO was followed by the tentOCSFO and sineOCSFO algorithms. The logisticOCSFO was followed by the tentOCSFO and sineOCSFO algorithms. The true minimum value of the sphere function is 0, and sineOCSFO and tentOCSFO provided the closest values to this. ClassicOCSFO and circleOCSFO were other chaotic-based algorithms that approached this value. tentOCSFO stood out in terms of the results it provided for the mean and median values. Furthermore, classicOCSFO yielded better results than the chebyshevOCSFO and logisticOCSFO algorithms among chaotic-based SFOs. Another function with a true minimum value of 0 is the Rosenbrock Disk function. All methods were quite successful and performed well for this function. Based on the lowest and highest values, classicSFO and tentOCSFO were slightly less efficient than the others. Although the algorithms were successful in reaching the minimum value in Rosenbrock Cubic/Line, chebyshevOCSFO showed relatively low performance for the mean and median. When all these results are evaluated together, it is seen that chaoticSFO can produce more competitive results compared to classicalSFO. Among chaoticOCSFO, tentOCSFO stands out compared to other algorithms. Although it generally produces successful results, chebyshevOCSFO lags behind other chaoticOCSFO.

Based on the competitive optimization performance observed in benchmark tests, the following subsection evaluates the effectiveness of OCSFO in quantitative rule mining for drying efficiency classification.

#### 3.2.2. Task-Specific Performance Evaluation of OCSFO

The flexibility of the algorithm used stems from the use of both chaotic and trigonometric approaches. The rules and metrics found by the proposed optimization algorithm according to the relevant classes are given in Table 9, Table 10 and Table 11. In this classification model, the data were determined as high (H), medium (M), and low (L) using the drying efficiency ranges in Table 4.

Furthermore, the consistency of accuracy values across various iterations suggests that the OCSFO algorithm delivers robust rule mining performance with minimal sensitivity to random initialization. The diminished accuracy values noted for the low-efficiency (L) class can be ascribed to the overlapping parameter ranges during the latter phases of drying, characterized by increased energy consumption and diminished moisture removal. The subsequent section examines the physical significance and interpretability of the extracted rules, despite the quantitative success of OCSFO in rule mining being demonstrated by these results.

### 3.3. Extracted Explainable Rules

In the study, the rules created using the oscillating chaotic sunflower optimization (OCSFO) classified drying efficiency as high (H), medium (M), and low (L). The fact that the accuracy rates of the obtained rules were above 90% indicates that the parameters are significantly related to drying efficiency.

*High efficiency (H):*

- Typically achieved under conditions such as an output temperature range of 37–49 °C;
- Product weight between 260–430 g;
- Heat energy ($Q_{U}$) produced varying between 3.5–10 W.

These rules indicate scenarios in which the product’s moisture is quickly removed and energy use is efficient. The literature also states that in hot-air dryers, moisture transfer accelerates within a certain temperature and air flow rate range, thus optimizing drying time and energy consumption [1,5].

*Medium efficiency (M):*

- Cabin interior temperature between 55–63 °C;
- Product weight should be around 260–310 g;
- Air velocity is at moderate values such as 0.69–0.76 m/s.

This situation indicates that the system is still functioning effectively during the drying process but is operating slightly outside optimal conditions. Similarly, Guiné et al. [4] have shown that efficiency decreases when drying temperatures deviate from optimal values.

*Low efficiency (L):*

- The output temperature must be within the range of 48–62 °C;
- Product weight decreasing to 100–300 g levels;
- The input temperature must remain between 29–40 °C.

In these scenarios, since the product is already largely dried, removing water has become more difficult, and the system’s energy consumption has increased while but efficiency has decreased. The literature also frequently reports that energy efficiency decreases and drying speed drops in the final stages of the drying process [6,8].

The resulting rules are consistent with general trends in the food drying literature. The parameters align meaningfully with both physical processes and findings reported in previous studies. Derived using explainable artificial intelligence, these rules go beyond black-box models, clearly showing researchers which parameter ranges yield high efficiency.

These rule-based interpretations demonstrate OCSFO’s ability to explain energy-efficient drying behaviors in a transparent and physically meaningful way.

## 4. Discussion

The derived explicable principles offer distinct insights into the correlation between operational factors and drying efficacy for “Paşa” pears. High drying efficiency is primarily linked to moderate to high air velocity and increased cabinet temperature ranges, aligning with the core principles of convective heat and mass transfer. Elevated air velocity facilitates moisture diffusion from the product surface, while increased drying temperatures augment the vapor pressure gradient, therefore expediting moisture removal during the first and intermediate phases of drying. Nonetheless, the regulations stipulate that excessive energy input during the final phases of drying does not inherently result in commensurate efficiency improvements. As moisture content declines, internal diffusion resistance prevails, resulting in declining returns from moisture extraction despite heightened energy expenditure. This pattern elucidates the diminished efficiency observed during the final drying phase and underscores the necessity of equilibrating thermal input and airflow to attain energy-efficient drying. The rule-based outcomes thus represent physically significant drying dynamics rather than merely data-driven correlations.

Most current research on food drying optimization depends on black-box machine learning techniques, such as artificial neural networks or deep learning frameworks, which attain great predictive accuracy but provide minimal interpretability. Although these models are proficient at prediction tasks, they offer less understanding of how certain operating parameters affect drying performance, thereby limiting their utility in practical decision-support systems. This paper presents a rule mining system driven by explainable artificial intelligence (XAI) that produces transparent, interpretable decision rules, clearly delineating parameter ranges linked to various efficiency levels. These rule-based explanations allow domain specialists to clearly comprehend the influence of air velocity, temperature, and energy-related variables on drying efficiency without necessitating supplementary post-hoc interpretation methods. In contrast to black-box methods, the suggested technique enhances knowledge extraction, process comprehension, and practical application, especially in industrial drying contexts where transparency and controllability are crucial.

The exceptional efficacy of the OCSFO algorithm in benchmark optimization assessments and task-specific rule mining is due to its improved balance between exploration and exploitation. By integrating oscillatory search behavior, OCSFO dynamically adjusts step sizes, facilitating efficient local refining around interesting solutions. The incorporation of chaotic mapping techniques enhances population variety and diminishes vulnerability to random initialization, thus alleviating premature convergence. These attributes are especially beneficial in quantitative rule-mining challenges, where intricate, nonlinear relationships among factors may result in fragmented or inefficient rule sets. The stability exhibited across numerous iterations indicates that OCSFO can reliably derive high-quality classification rules with robust generalization ability. The approach enhances optimization robustness and facilitates the creation of compact, diversified rule sets suitable for explainable artificial intelligence applications.

Despite the promising results obtained in this study, several limitations should be acknowledged. The experimental investigation focused solely on the “Paşa” pear, processed with a fixed slice thickness and a conventional hot-air drying setup. The extracted rules have clear physical meaning and internal consistency. However, these rules cannot be directly applied to other food materials, geometries, or drying technologies without further validation. The drying experiments also covered a limited range of air temperature and velocity, selected to maintain process stability and product quality. This limited scope may limit how widely the derived rules can be used across different industrial conditions. The proposed rule-based framework mainly relies on thermal, mass-related, and energy-based sensor data. It does not explicitly incorporate visual attributes linked to product quality, such as color changes, surface shrinkage, or texture. The OCSFO-based rule mining procedure was performed offline. Integrating it directly into a real-time adaptive control loop was beyond the scope of this study. Future research will extend this methodology to different food products and drying methods. The XAI framework will be enriched with image-based features and real-time, OCSFO-driven rules. These improvements aim to enable fully autonomous, energy-efficient drying systems.

## 5. Conclusions

This study involved the drying of “Paşa” pears, a regional agricultural product from Elazig, Turkiye, under various operational conditions utilizing an intelligent hot-air drying system. Experimental investigations were performed at two air velocity levels (0.63 m/s and 1.03 m/s) and two drying temperatures (50 °C and 65 °C) to assess drying behavior, energy efficiency, and process performance.

The most rapid drying process occurred at an air velocity of 1.03 m/s and a temperature of 65 °C, resulting in a reduction of product weight from roughly 450 g to 103 g. The slowest drying transpired at an air velocity of 0.63 m/s at a temperature of 50 °C. The optimal drying efficiency, from an energy efficiency standpoint, was achieved at an air velocity of 0.63 m/s at a temperature of 65 °C, yielding a value of 8.57%. Conversely, the minimal efficiency was noted at 3.65% with the same air velocity of 0.63 m/s but at a temperature of 50 °C. The results demonstrate that expedited drying does not inherently equate to enhanced energy efficiency, underscoring the significance of balanced operational conditions.

A quantitative rule mining framework based on explainable artificial intelligence (XAI) was utilized to facilitate transparent and interpretable analysis. In this context, the oscillating chaotic sunflower optimization (OCSFO) algorithm, an optimization algorithm inspired by plant intelligence, was employed for the first time in food drying applications to derive classification rules for high, medium, and low efficiency drying conditions. The suggested methodology effectively demonstrated distinct, physically significant correlations between drying factors and energy efficiency, with the derived classification rules attaining accuracy rates beyond 90%. The extracted rules indicated that drying temperature and air velocity are the primary determinants of drying efficiency, but energy consumption and inside cabinet temperature distribution serve a supplementary role in differentiating efficiency classes. Three typical rules were produced for each efficiency class, ensuring interpretability and robustness.

The findings indicate that the devised intelligent drying system, in conjunction with the suggested XAI-driven rule mining methodology, facilitates optimal energy consumption while maintaining product quality. To guarantee a physically significant and resilient classification, average drying efficiency was selected as the goal variable rather than instantaneous efficiency, offering a stable system-level performance metric appropriate for optimization-based rule extraction. This research develops a clear, data-informed decision-support framework for intelligent and energy-efficient food drying applications. Future endeavors will concentrate on augmenting the proposed methodology by including image processing techniques into the control framework and formulating adaptive real-time control strategies to further improve autonomous drying efficacy.

Future work will focus on developing XAI-based rule models for different food products (e.g., peppers) and integrating these rules with deep learning-based image processing to implement smart, energy-efficient control strategies for autonomous food drying systems.

## Figures and Tables

**Table 1:** Hardware list.

ID	Hardware	ID	Hardware
1	Drying cabinet	8	Light Emitting Diode (LED) lighting
2	Laser temperature sensor	9	Heater
3	Camera	10	Programmable Logic Controller (PLC) automation unit
4	Load cell	11	Ambient temperature sensor
5	Air velocity sensor	12	Axial fan
6	Drying tray	13	Exhaust flap and motor
7	Temperature–humidity sensor		

**Table 2:** Uncertainty values of measurement parameters.

Parameter	Sensor	Brand	Unit	Uncertainty
Air Temperature	Thermohygrometer	Belimo/Switzerland	°C	%1.8
Air Speed	Velocity transmitter	Siemens/Germany	m/s	±0.05 m/s
Weight	Loadcell	Delta/Taiwan	gram	±0.1 g
Energy Consumption	Energy analyzer	Uni-T/China	joule	%1.4

**Table 3:** Parameters for explainable artificial intelligence rules.

Parameters	Unit	Symbol	Max. Value	Min. Value
Total energy consumption (fi_Cons) (Fan + Heater)	Watt	$E_{T}$	450	325
Air speed (air_speed)	m/s	V	1.03	0.63
Drying cabinet internal temperature (cabinet_temp)	°C	$T_{C}$	65	50
Heat energy produced (qu)	Watt	$Q_{U}$	11.55	7.18
Cabinet inlet temperature (input_tmp)	°C	$T_{I}$	41.90	29.60
Cabinet outlet temperature (out_temp)	°C	$T_{O}$	66.60	39.60
Weight (weight)	Gram	G	450.10	102.90
Drying efficiency (H (High)/M (Medium)/L (Low))	%	$\eta_{d}$	33.60	0.63

**Table 4:** Class metrics defined for drying efficiency.

Drying Efficiency Range	Description
$35>\eta_{d}\geq 20$	High drying efficiency
$20>\eta_{d}\geq 10$	Medium drying efficiency
$10>\eta_{d}>0$	Low drying efficiency

**Table 5:** Calculation of TP, TN, FP, and FN values.

“If” Condition	“Then” Condition	Operation
True	True	Increment TP by 1
False	False	Increment TN by 1
True	False	Increment FP by 1
False	True	Increment FN by 1

**Table 6:** Selected chaotic functions (maps).

Name	Functions	Parameters
Sine Function	$X_{n+1}=\frac{a}{4}sin(\pi X_{n})$	0<a≤4
Logistic Function	$X_{n+1}=\mu X_{n}(1-X_{n})$	0<μ≤4
Chebyshev Function	$X_{n+1}=cos(k{cos}^{-1}X_{n})$	k≥2
Circle Function	$X_{n+1}=X_{n}+0.2-\left(\frac{1}{4\pi }\right)sin(2\pi X_{n})mod(1)$	
Tent Function	$X_{n+1}=\left\{\begin{array}{c} \mu X_{n},\;\;\;\;\;\;\;\;\;\;\;\;\;X_{n}<\frac{1}{2}\\ \mu \left(1-X_{n}\right),\;\;\;\frac{1}{2}\leq X_{n} \end{array}\right\}$	

**Table 7:** Quality test functions used in experiments.

Name	Equation	Constraint	Minimum
Sphere	$f\left(x\right)=\sum_{i=1}^{N}x_{i}^{2}$	$-100<x_{i}<100$	0
Rastrigin	$f\left(x\right)=10N+\sum_{i=1}^{N}\left[x_{i}^{2}-10cos(2\pi x_{i})\right]$	$-5.12<x_{i}<5.12$	0
Camel	$f\left(x\right)=\left(4-2.1x_{1}^{2}+\frac{x_{1}^{4}}{3}\right)x_{1}^{2}+x_{1}x_{2}+(-4+4x_{2}^{2})x_{2}^{2}$	$-200<x_{i}<200$	−1.031
Nonlinear	$f\left(x\right)=x_{1}^{2}-3x_{1}x_{2}+4x_{2}^{2}+x_{1}-x_{2}$	$-2<x_{i}<2$	−0.28
Rosenbrock Cubic/Line	$f\left(x,y\right)={\left(1-x\right)}^{2}+100{\left(y-x^{2}\right)}^{2}$	${\left(x-1\right)}^{3}-y+1\leq 0$ and x+y−2≤0	0
Rosenbrock Disk	$f\left(x,y\right)={\left(1-x\right)}^{2}+100{\left(y-x^{2}\right)}^{2}$	$x^{2}+y^{2}\leq 2$	0

**Table 8:** Summary results for independent OCSFO experiments.

		**Camel**	**Nonlinear**	**Rastrigin**	**Sphere**	**Disk**	**Cubic/Line**
Classic	Min	−1.03	−0.29	1.47	0.00	0.00	0.00
	Max	−1.03	−0.29	12.05	1.03	0.03	0.00
	Average	−1.03	−0.29	6.42	0.09	0.00	0.00
	Median	−1.03	−0.29	6.48	0.02	0.00	0.00
	Std. Deviation	0.00	0.00	3.01	0.23	0.01	0.00
Chebyshev	Min	−1.03	−0.29	3.55	0.01	0.00	0.00
	Max	−1.03	−0.29	33.34	6.49	0.00	6.78
	Average	−1.03	−0.29	17.78	0.89	0.00	0.76
	Median	−1.03	−0.29	17.57	0.40	0.00	0.00
	Std. Deviation	0.00	0.00	7.97	1.41	0.00	1.86
Circle	Min	−1.03	−0.29	2.67	0.00	0.00	0.00
	Max	−0.22	−0.29	17.73	1.14	0.00	0.00
	Average	−0.99	−0.29	5.68	0.14	0.00	0.00
	Median	−1.03	−0.29	4.79	0.07	0.00	0.00
	Std. Deviation	0.18	0.00	3.50	0.25	0.00	0.00
Logistic	Min	−1.03	−0.285	0.89	0.00	0.00	0.00
	Max	−1.03	−0.285	11.40	1.33	0.00	0.95
	Average	−1.03	−0.29	4.70	0.33	0.00	0.07
	Median	−1.03	−0.29	4.03	0.12	0.00	0.00
	Std. Deviation	0.00	0.00	3.30	0.39	0.00	0.22
Sine	Min	−1.03	−0.29	1.03	0.00	0.00	0.00
	Max	−1.03	−0.29	11.04	2.44	0.01	3.21
	Average	−1.03	−0.29	4.26	0.24	0.00	0.16
	Median	−1.03	−0.29	3.66	0.03	0.00	0.00
	Std. Deviation	0.00	0.00	2.59	0.54	0.00	0.70
Tent	Min	−1.03	−0.29	1.01	0.00	0.00	0.00
	Max	−1.03	−0.29	10.91	0.29	0.00	0.11
	Average	−1.03	−0.29	3.91	0.04	0.00	0.01
	Median	−1.03	−0.29	3.49	0.01	0.00	0.00
	Std. Deviation	0.00	0.00	2.36	0.07	0.00	0.03

**Table 9:** Experiments conducted for Class H.

**Experiment-1**	
**Rule**	**Accuracy**
if 34.725 < out_temp < 47.526 and 260.569 < weight < 448.000 then H	0.906
if 3.457 < qu < 7.678 then H	0.906
if 4.688 < qu < 7.663 then H	0.875
if 39.352 < out_temp < 49.398 then H	0.875
**Experiment-2**	
**Rule**	**Accuracy**
if 29.650 < input_tmp < 39.162 and 38.432 < out_temp < 47.802 then H	0.875
if 3.914 < qu < 9.254 and 37.440 < out_temp < 58.178 then H	0.875
if 37.648 < out_temp < 48.590 then H	0.875
if 32.207 < input_tmp < 38.608 and 43.250 < out_temp < 49.740 then H	0.844
**Experiment-3**	
**Rule**	**Accuracy**
if 4.449 < qu < 7.310 and 207.437 < weight < 433.666 then H	0.875
if 341.240 < weight < 415.963 then H	0.875
if 3.457 < qu < 8.523 and 29.675 < input_tmp < 34.916 then H	0.875
if 5.025 < qu < 9.629 and 46.411 < out_temp < 52.462 then H	0.844
**Experiment-4**	
**Rule**	**Accuracy**
if 3.481 < qu < 10.128 and 313.120 < weight < 448.000 then H	0.875
if 3.490 < qu < 8.900 and 29.758 < input_tmp < 38.685 then H	0.875
if 3.457 < qu < 9.129 and 29.650 < input_tmp < 37.606 then H	0.875
if 6.059 < qu < 8.839 and 41.149 < out_temp < 50.211 then H	0.844
**Experiment-5**	
**Rule**	**Accuracy**
if 29.650 < input_tmp < 34.492 and 37.000 < out_temp < 49.069 and 105.200 < weight < 430.153 then H	0.875
if 25.796 < out_temp < 48.131 then H	0.906
if 37.000 < out_temp < 48.989 then H	0.906
if 3.572 < qu < 10.469 and 305.708 < weight < 409.503 then H	0.875

**Table 10:** Experiments conducted for Class M.

**Experiment-1**	
**Rule**	**Accuracy**
if 341.456 < fi_Cons < 372.798 and 60.945 < cabinet_temp < 63.775 then M	0.938
if 275.570 < weight < 302.632 then M	0.938
if 263.973 < weight < 305.169 then M	0.938
if 54.454 < cabinet_temp < 61.180 and 5.729 < qu < 11.877 and 313.348 < weight < 404.184 then M	0.938
**Experiment-2**	
**Rule**	**Accuracy**
if 0.693 < air_speed < 0.765 then M	0.938
if 30.111 < input_tmp < 36.403 and 268.232 < weight < 293.215 then M	0.938
if 327.102 < fi_Cons < 408.835 and 50.171 < cabinet_temp < 60.107 then M	0.938
if 413.098 < fi_Cons < 436.363 and 46.160 < out_temp < 53.450 then M	0.938
**Experiment-3**	
**Rule**	**Accuracy**
if 395.812 < fi_Cons < 414.856 and 53.807 < cabinet_temp < 59.877 and 4.954 < qu < 8.258 and 420.508 < weight < 440.947 then M	0.938
if 1.005 < air_speed < 1.008 and 10.328 < qu < 12.019 and 37.126 < out_temp < 54.434 then M	0.938
if 0.825 < air_speed < 0.836 and 30.913 < input_tmp < 40.210 and 44.608 < out_temp < 44.895 then M	0.938
if 50.039 < cabinet_temp < 64.807 and 8.138 < qu < 9.550 and 45.784 < out_temp < 47.581 then M	0.938
**Experiment-4**	
**Rule**	**Accuracy**
if 268.524 < weight < 317.923 then M	0.938
if 64.438 < cabinet_temp < 64.454 and 32.141 < input_tmp < 39.354 then M	0.938
if 0.756 < air_speed < 0.848 and 165.000 < weight < 323.900 then M	0.938
if 52.585 < cabinet_temp < 57.295 and 8.664 < qu < 10.176 then M	0.938
**Experiment-5**	
**Rule**	**Accuracy**
if 285.052 < weight < 310.401 then M	0.938
if 288.029 < weight < 309.230 then M	0.938
if 7.903 < qu < 11.557 and 42.390 < out_temp < 57.200 and 256.262 < weight < 258.360 then M	0.938
if 8.069 < qu < 8.367 and 42.320 < out_temp < 59.132 and 286.273 < weight < 422.934 then M	0.938

**Table 11:** Experiments conducted for Class L.

**Experiment-1**	
**Rule**	**Accuracy**
if 48.350 < out_temp < 57.233 then L	0.875
if 29.662 < input_tmp < 37.372 and 47.581 < out_temp < 61.746 then L	0.844
if 7.602 < qu < 12.179 and 29.650 < input_tmp <40.096 then L	0.844
if 29.690 < input_tmp < 34.545 and 48.394 < out_temp < 62.496 then L	0.844
**Experiment-2**	
**Rule**	**Accuracy**
if 46.921 < out_temp < 62.586 then L	0.875
if 29.650 < input_tmp < 39.818 and 47.391 < out_temp < 55.985 then L	0.844
if 29.650 < input_tmp < 40.210 and 49.055 < out_temp < 61.495 then L	0.844
if 29.710 < input_tmp < 35.734 and 40.287 < out_temp < 59.367 then L	0.844
**Experiment-3**	
**Rule**	Accuracy
if 46.939 < out_temp < 61.434 then L	0.875
if 47.870 < out_temp < 62.603 then L	0.875
if 29.693 < input_tmp < 36.442 and 51.003 < out_temp < 56.194 then L	0.844
if 29.720 < input_tmp < 36.057 and 48.071 < out_temp < 57.444 then L	0.844
**Experiment-4**	
**Rule**	**Accuracy**
if 46.685 < out_temp < 57.868 then L	0.875
if 48.949 < out_temp < 59.674 then L	0.875
if 48.890 < out_temp < 61.905 then L	0.875
if 29.650 < input_tmp < 32.956 and 46.011 < out_temp < 62.620 then L	0.844
**Experiment-5**	
**Rule**	**Accuracy**
if 29.664 < input_tmp < 39.317 and 47.754 < out_temp < 62.408 then L	0.844
if 47.084 < out_temp < 59.303 then L	0.875
if 37.403 < out_temp < 55.636 then L	0.844
if 26.338 < input_tmp < 33.271 and 100.876 < weight < 300.821 then L	0.844

## Data Availability

The data presented in this study are available from the corresponding author upon reasonable request.
